# Case Report: Creutzfeldt-Jakob disease and diagnosis challenges: case report and evidence synthesis

**DOI:** 10.12688/f1000research.150498.2

**Published:** 2025-12-09

**Authors:** Virgilio E Failoc Rojas, Yiro Yazawa-Ballena, Gustavo Alvarado-Moreno, Alba Navarro-Flores, Carlos Alva-Diaz, Kevin Pacheco-Barrios

**Affiliations:** 1 Universidad Cesar Vallejo, Piura, Piura, 20001, Peru; 2 Internal medicine, Hospital Regional Lambayeque, Lambayeque, Lambayeque, 14011, Peru; 3 International Max Planck Research School for Translational Psychiatry (IMPRS-TP), Munich, Germany, 80804, Germany; 4 Institute of Psychiatric Phenomics and Genomics (IPPG), LMU University Hospital, LMU Munich, Munich, Germany; 5 Servicio de Neurología, Departamento de Medicina y Oficina de Apoyo a la Docencia e Investigación (OADI), Hospital Daniel Alcides Carrión, Callao, Peru; 6 Grupo de Investigación NEMECS: Neurociencias, Metabolismo, Efectividad Clínica y Sanitaria, Universidad Cientifica del Sur, Lima, Peru; 7 Universidad San Ignacio de Loyola, Lima District, Lima Region, 150114, Peru

**Keywords:** Creutzfeldt-Jakob syndrome; Prion diseases; differential diagnosis; behavioral symptoms; 14-3-3 Proteins; Peru

## Abstract

**Introduction:**

Prion diseases are mortal neurodegenerative disorders, which include Creutzfeldt-Jakob disease (CJD). Due to its heterogenous clinical presentation diagnosis uncertainties are common. In this paper we explore CJD diagnostic challenges focusing on differential diagnosis and diagnostic delays.

**Methods:**

We report a case of a patient who was misclassified and evaluated by several medical specialties before the CJD suspicion. A systematic review of the literature of the CJD case reports focused on the timely and differential diagnosis was carried out in Medline and Embase until May 2023.

**Results:**

Patient with diagnosis was made due to the form of presentation and clinical evolution, neuroimaging and the presence of protein 14-3-3. In systematic review, fifteen articles were selected, who reported 31 cases of CJD with problems in the timely diagnosis and incorrect initial diagnosis, the main initial differential diagnoses were psychiatry exacerbation, myelopathy, epilepsy, stroke, parkinsonism, cerebellar ataxia and autoimmune encephalitis. The most common clinical onset was psychobehavioral disturbances (apathy, confusion and sleep disturbance), extrapyramidal signs and cognitive impairment. The diagnosis delay was from one to eighteen months.

**Conclusion:**

A discussion of the case report and the diagnostic challenges reported in the literature was made. Patients can present a wide range of symptoms. It is recommended to consider CJD for the differential diagnosis in patients with behavioral symptoms, and cognitive impairment.

## Introduction

Creutzfeldt-Jakob disease (CJD) is one of the main prion diseases, an always fatal neurodegenerative disorder. ^1^ The common pathological process is characterized by the conversion of the normal cellular prion protein (PrPc) to an insoluble anomalous form (PrPSc) that accumulates progressively in the brain, sometimes forming extracellular amyloid plaques. ^2^

CJD is classified, according to the etiology, into sporadic (idiopathic or classic) (85%), acquired (or exogenous) (5%), genetic (or hereditary) (10%), and other variants such as Gerstmann-Sträussler-Scheinker disease (GSSD), fatal familial insomnia (FFI) and kuru. ^1, 3, 4^

The probable diagnosis is based on progressive dementia and other clinical criteria (myoclonus, visual disorders, cerebellar signs, pyramidal or extrapyramidal signs, and akinetic mutism), as well as a compatible electroencephalogram (EEG) (periodic epileptiform discharges) and/or detection of neuronal protein related to neuronal destructions that accumulates in cerebrospinal fluid (CSF), called protein 14-3-3. ^1, 3, 5^ The definitive diagnosis requires a post-mortem study. ^6^

Due to these clinical complexities and the variable initial onset, it presents a common delayed detection with issues during the differential diagnosis. In this paper, we include a representative case from our institution and discuss the common challenges during the course of the disease.

We present a case report from the “Hospital Regional Lambayeque”, in Lambayeque, Peru. This report followed the CARE guidelines. ^7^

### Consent

Written informed consent for publication of their clinical details and/or clinical images was obtained from the patient.

## Case report

We present the case of a 47-year-old male from the Amazon region of Peru, unemployed, with no pathological or surgical history. His symptoms start with oppressive non-located headache 80-weeks before, which were treated with analgesic medication without response. It was associated with a hands rest tremor, perceptual alteration of time, difficulty in recognizing relatives, aggressiveness, agitation, and mild memory impairment.

24-weeks before, symptomatology persists and progress until presenting alterations of sphincter control and difficulty for walking, and upper limb rest and intention tremor generating partial dependence for their daily activities. On detailed neurological examination, the patient presented both extrapyramidal (rest and intention tremor, and bradykinesia) and pyramidal signs (generalized hyperreflexia and bilateral Babinski sign). Gait instability with intention tremor suggested cerebellar involvement, and intermittent involuntary jerks observed during hospitalization were compatible with early myoclonus. He had a hospital admission for presenting focal to bilateral tonic-clonic seizures for which he received antiepileptic drugs with an incomplete response. Subsequently, due to the persistence of symptomatology and the development of epileptic status, he was hospitalized again. The blood studies showed anemia (10.2 g/dl), with any other abnormalities. The CSF analysis found a clear fluid without alterations.

**Figure 1.**
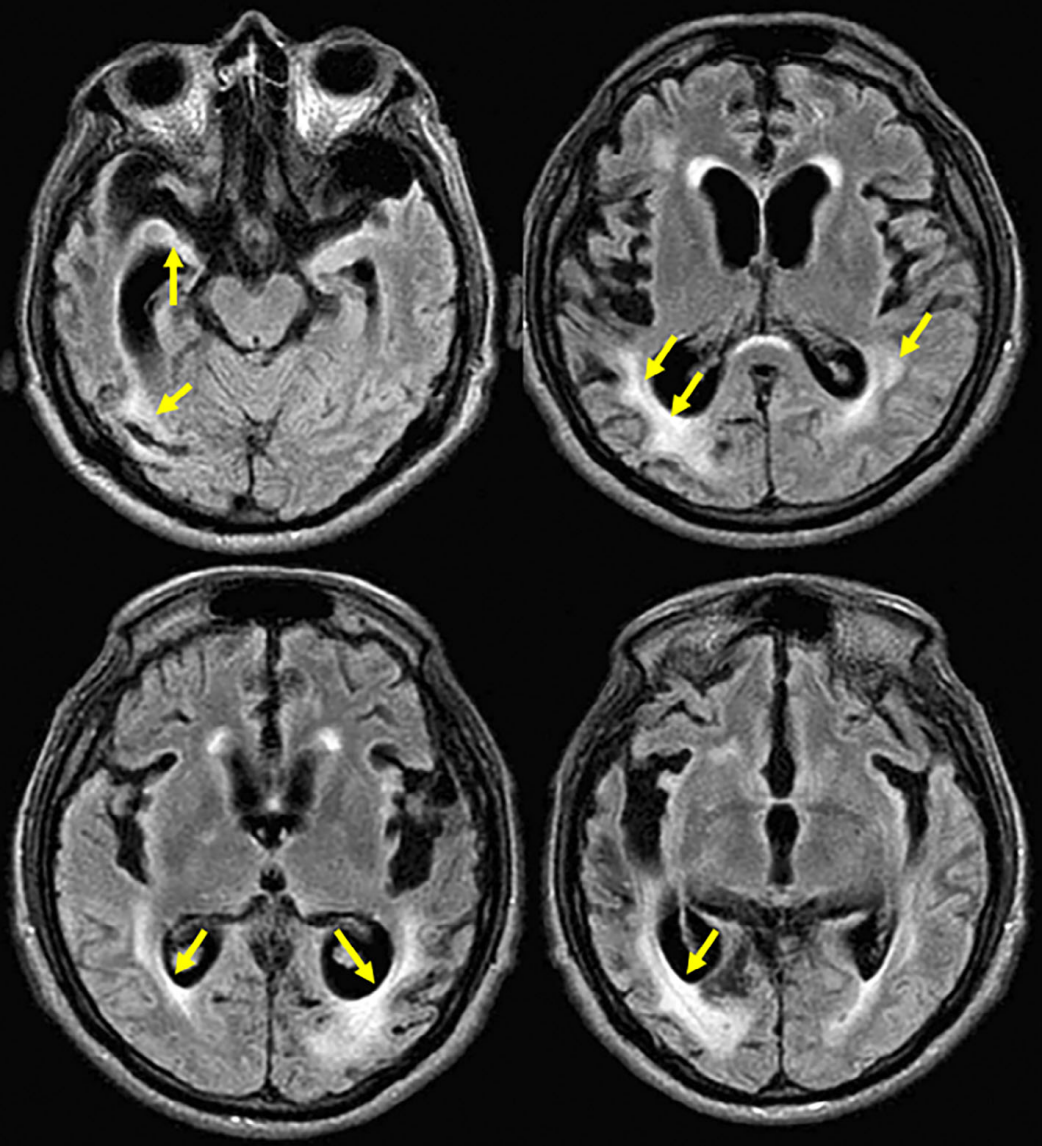
Axial brain-MRI of the patient with probable sporadic CJD. Axial section in a fluid attenuated inversion recovery (FLAIR) sequence showing hippocampal volume decreased in both temporal lobes with increase of temporal horns predominantly on right side; mild hyperintensity in right caudate nucleus and moderate in periventricular regions with confluent zones in the right parietal region; and moderate to severe atrophic cortical-subcortical with frontotemporal predominance (Hyperintensities highlighted with arrows).

Brain magnetic resonance imaging (MRI) showed moderate to severe cortico-subcortical atrophic changes and subcortical hyperintensities ( Figure 1). Additionally, an EEG was performed, without any alteration. Due to the progressive neurological compromise and the combination neurological findings, patient met the requirement of at least two supportive signs (pyramidal/extrapyramidal and cerebellar/myoclonic features) as defined in the diagnostic criteria for probable CJD. Moreover, the development of early and progressive dementia in the next months, and the MRI suggestive of prion disease, CJD diagnostic was prioritized for exploration. CSF 14-3-3 protein was analyzed by a semi-quantitative Western blot assay in an external reference laboratory. Patient CSF was run alongside negative and positive control samples, and band intensity for 14-3-3 was densitometrically compared to these controls. According to the laboratory’s validated cut-off, the result was classified as strongly positive (‘high titre’), supporting the diagnosis of probable CJD. Symptomatic treatment and palliative care were indicated to the patient.

All these tests were performed during the hospitalization of the patient. The Western blot test of the protein 14-3-3 was performed two weeks after his hospitalization. The patient was evaluated one month after discharge, but he did not return for his next consultation, and it was not possible to follow up on his case.

## Discussion

The case reported a progressive neurological condition (headache that progress to dementia) with early onset of cognitive impairment and behavioral symptoms at the age of 47 years, a Brain-MRI showing moderate to severe cortico-subcortical trophic changes, and basal nuclei hyperintensity, added to a positive Western-Blot quantification of CSF 14-3-3 protein (sensitivity of 94% and specificity of 84%), ^5, 8^ hence the condition was classified as spongiform encephalopathy, probable CJD. It was not possible to make the definitive diagnosis with brain biopsy confirmation, where neuronal loss, gliosis, and intracytoplasmic vacuolization are observed in the brain parenchyma. ^6, 9^

CJD is a rare and fatal neurodegenerative disease, due to the high mortality, an early and timely diagnosis must be made. ^10, 11^ However, currently the diagnostic criteria are controversial and there are several variances of the onset symptoms, thus, the diagnosis is usually made in the terminal phases of the disease.

We search the available literature to compare with our experience and found ten articles reporting diagnostic challenges in CJD ( Table 1). ^12– 21^ The age range of the patients reported was 30 to 86 years [median 64 years]. The main initial clinical manifestation was psycho-behavioral symptoms (50% of the cases), predominantly: apathy, confusion, aggressiveness, irritability, and sleep disturbance. The initial differential diagnoses were psychiatry exacerbation (depression and schizophrenia), myelopathy, epilepsy, stroke and parkinsonism. Our patient is 47-years old, male, with an initial presentation of headache, extrapyramidal signs, and behavioral symptoms. In another case series, ^22^ they found that the presentation was nonspecific, from headache to ataxias. The extrapyramidal and cerebellar signs onset is presented as a frequent initial manifestation. ^23^ These data coincide with our systematic review results, indicating a common non-specific initial symptomatology in these cases.

**Table 1.** Description of previously reported cases with diagnostic challenges.

Author (year)	Patient information	Clinical manifestations	Initial diagnosis	Neuroimaging	EEG	Differential diagnosis	Time until final diagnosis	Final diagnosis
Ziso, B. et al (2017)	57-years-old woman, no previous diseases history.	1-month history of sensorial disturbances in left arm and walk difficulties. Hypertonia and hyperreflexia. 5 weeks after worsening of mobility, quadriparesis, and sphincter dysfunction.	Myelopathy	Restricted diffusion in the right frontal cortical region.	Theta range with slower delta rhythms.	Cervical radiculopathy	6 weeks	Sporadic CJD (clinical + 14-3-3 protein in CSF)
Roest et al. (2016)	51-years-old woman, depression history.	Suicide ideation, memory impairment and sleep disturbances. 14 weeks after worsening cognitive impairment and confusion.	Depression exacerbation	Normal	Initial: w/o alterations. After 14 weeks: generalized, synchronous triphasic complexes.	Depression and posttraumatic stress disorder.	14 weeks	Sporadic CJD (clinical + 14-3-3 protein in CSF)
Ghadiri-Sani et al. (2015)	56-years old woman, with history of stroke 10 years ago.	Left homonymous hemianopia, dysarthria, mild left-sided weakness, hyperreflexia with bilateral Babinski. After 4 weeks: progressive episodes of confusion and apparent blindness, cognitive deterioration.	Multiple territory strokes	Initial: w/o alterations. After 4 weeks: mild global brain atrophy was noted.	Triphasic waves	Vertebral artery dissection with showers of emboli or cerebral vasculitis.	5 weeks	Sporadic CJD (Biopsy)
Chauvin et al. (2014)	78-years-old man, no previous diseases history.	Tonic-clonic seizures. 3-months after: muscular weakness. 4-months after: progressive cognitive impairment (apraxia and akinetic mutism).	Epilepsy	Initial: w/o alteration. After 3 months: Fronto-temporal hypersignal.	Normal	Myelopathy	15 weeks	Sporadic CJD (clinical + 14-3-3 protein in CSF)
Damato et al. (2014)	78 years-old man, hypertension history.	Right hemiparesis, Babinski sign present and dysarthria. After 4 weeks: intermittent myoclonic jerks in both arms and legs and a rapidly progressive cognitive decline and abnormal behavior, confusion and agitation.	Stroke	Showed hyperintensity of left frontal gyri on T2-weighted FLAIR.	Diffuse theta -delta activity.	Stroke mimics	5 weeks	Sporadic CJD (clinical + 14-3-3 protein in CSF)
Jacquin et al. (2014)	64-years-old man, no previous diseases history.	Language disorders, apathy and behavioral disorders.	Progressive primary aphasia	Not reported	Not reported	CJD	Not reported	Sporadic CJD (clinical + 14-3-3 protein in CSF)
	68-years-old man, no previous diseases history.	Aphasia, apraxia and ataxia of right upper limb with parkinsonian syndrome.	Corticobasal degeneration	Not reported	Not reported	Parkinsonism	Not reported	Sporadic CJD (clinical + 14-3-3 protein in CSF)
	2 Case reports, females, 58 and 61 years-old, no previous diseases history.	Anxiety and depression syndrome, falls, visual hallucinations, extra-pyramidal syndrome and fluctuating cognitive decline.	Lewy body dementia	Not reported	Not reported	CJD	Not reported	Sporadic CJD (clinical + 14-3-3 protein in CSF)
Chuang et al. (2012)	61 years-old man, history of schizophrenia.	Initial: visual hallucinations, hyperreflexia and parkinsonism. After 4 days: multifocal myoclonus. After 1 month: cognitive decline and behavior change.	Exacerbation of schizophrenia	After 8 days: Hyperintensities on diffusion weighted imaging in the occipital lobes bilaterally.	After 6 days: left periodic lateralizing epileptiform discharges (PLEDs), generalized disorganization, and slowing.	Parkinsonism	4 weeks	Sporadic CJD (clinical + 14-3-3 protein in CSF)
Mahmoudi et al. (2010)	74-year-old man, no previous diseases history.	Psychobehavioral disturbances including verbal aggressiveness, anxiety, and irritability. After 18 months: cognitive and psychobehavioral symptoms increased, and gait disorders appeared.	Alzheimer disease	Cortico-subcortical atrophy	Not reported	Atypical AD	72 weeks	Sporadic CJD (clinical + 14-3-3 protein in CSF)
Chadenat et al. (2009)	79-years-old woman, no previous diseases history.	Subacute dementia associated with gait disorder. Serum calcium elevated.	CJD	Not reported	Not reported	Metabolic-originated cognitive impairment	Not reported	Creutzfeldt-Jakob like syndrome due to parathyroid Adenoma
duPlessis et al. (2008)	74-year-old man, no previous diseases history.	Cognitive impairment and visual hallucination and parkinsonism. After 18 months: rapid cognitive decline and orthostatic hypotension	Dementia with Lewy bodies	Not reported	Not reported	CJD	72 weeks	Sporadic CJD (Biopsy)
Hamaguchi et al. (2005)	Case 1: 65-year-old woman	Dementia, pyramidal signs, insomnia.	CJD	Not reported	Not reported	Dementia	Not reported	Sporadic CJD (clinical + 14-3-3 protein in CSF)
	Case 2: 75-year-old woman	Psychiatric symptoms, dementia, myoclonus	CJD	Not reported	Not reported	Major depression	Not reported	Sporadic CJD (clinical + 14-3-3 protein in CSF)
	Case 3: 65-year-old man	Dementia, myoclonus, cerebellar ataxia, akinetic mutism	CJD	Not reported	Not reported	Cerebellar ataxia	Not reported	Sporadic CJD (clinical + 14-3-3 protein in CSF)
	Case 4: 49-year-old woman	Insomnia, dementia, psychiatric symptoms, pyramidal signs, extrapyramidal signs, autonomic symptoms, myoclonus, akinetic mutism	Progressive supranuclear palsy	Not reported	Not reported	Dementia	Not reported	Sporadic CJD (clinical + 14-3-3 protein in CSF)
	Case 5: 64-year-old man	Visual symptoms, extrapyramidal signs, dementia, autonomic symptoms, psychiatric symptoms, myoclonus, akinetic mutism	Progressive supranuclear palsy	Not reported	Not reported	Dementia	Not reported	Sporadic CJD (clinical + 14-3-3 protein in CSF)
	Case 6: 30-year-old woman	Visual symptoms, psychiatric symptoms, cerebellar ataxia, dementia, pyramidal signs, extrapyramidal signs, myoclonus, akinetic mutism	Spinocerebellar degeneration	Not reported	Not reported	Dementia	Not reported	Sporadic CJD (clinical + 14-3-3 protein in CSF)
	Case 7: 71-year-old man	Cerebellar ataxia, autonomic symptoms, dementia	Spinocerebellar degeneration	Not reported	Not reported	Cerebellar ataxia	Not reported	Sporadic CJD (clinical + 14-3-3 protein in CSF)
	Case 8: 58-year-old man	Dementia, cerebellar ataxia, myoclonus, pyramidal signs, psychiatric symptoms	Alzheimer disease	Not reported	Not reported	Dementia	Not reported	Sporadic CJD (clinical + 14-3-3 protein in CSF)
Pereira et al. (2002)	63-year-old man, no previous diseases history.	Progressive memory loss and word finding difficulty	Alzheimer disease	Large areas of increased signal in the frontal, temporoparietal and occipital lobes in DWI.	Normal	CJD	32 weeks	Sporadic CJD (Biopsy)
Walsh et al. (2001)	76-year-old woman, history of multiple stroke.	Memory impairment and behavioral changes	Vascular dementia	Brain Atrophy, older stroke lesions.	Not reported	CJD	4 weeks	Sporadic CJD (clinical + 14-3-3 protein in CSF)
Seipelt et al. (1999)	7 Case reports, females, 44-86 years-old, history of Hashimoto’s thyroiditis	Progressive cognitive impairment, myoclonus, ataxia, and personality change or psychotic phenomena	CJD	Not reported	Not reported	autoimmune encephalitis	Not reported	Autoimmune encephalitis (Hashimoto’s encephalitis)
Wilhelm-gling et al. (1998)	58-year-old woman, history of Hashimoto’s thyroiditis	Unconsciousness, progressive severe dementia and generalized myoclonus. Increased thyroid antibodies titles.	CJD	Not reported	Slowing activity and episodes of triphasic waves.	Infectious encephalitis, autoimmune encephalitis	Not reported	Autoimmune encephalitis (Hashimoto’s encephalitis)

The initial neuroimaging evaluations were reported in nine of the cases, ^12– 14, 16– 21^ mainly, they found hyperintensity in frontotemporal lobes and cortico-subcortical atrophy. EEG evaluations reporting theta range with slower delta rhythms.

The broad clinical findings spectrum included a progressive neuropsychiatric syndrome –whose initial symptoms could be depression, insomnia, anxiety, apathy, and hallucinations. Besides, movement disorders– cerebellar ataxia, involuntary movements, myoclonus, chorea, and dystonia- and persistent pain or paresthesia, could be associated. Finally, dementia and akinetic mutism could be presented at the end of this disease.

In general, psychiatric symptoms precede neurological manifestations. ^9, 22, 24^ We found that 65% of cases start clinical manifestation with psychobehavioral symptoms; it is according to our case report.

Timely diagnosis of CJD is often difficult, in part because of the frequency of unusual variants, with potential modifications due to early interventions. The available literature on this topic shows a wide diagnosis delay range from four to 80 weeks [median 14 (5-72 weeks)]. The best approach is to add CJD as our differential diagnosis options, not only in the classical rapid-progressive dementia diagnosis, but also in front of patients with suspicious of psychiatry exacerbation, myelopathy, epilepsy, stroke, and Parkinsonism.

The reports show that autoimmune encephalitis due to Hashimoto’s thyroiditis and a CJD-like syndrome due to parathyroid adenoma could simulate the CJD clinical manifestations even meeting partially the diagnostic criteria. ^25^ Thus, it is important to evaluate carefully the autoimmune disease history in patients with CJD suspicious.

Current reviews indicate that the changes in brain-MRI are bilateral, symmetric, and predominant in basal ganglia and cortical regions, ^6, 26^ in addition, 85% show signs of atrophy in the MRI. ^6^ Our case findings are according to this literature, however, in most of the cases with difficult timely diagnosis, the MRI results were inconclusive or normal until the end-stage of the disease, and the most common finding was cortical-subcortical atrophy and basal nuclei hyperintensity.

The patient presented unspecific characteristics of onset, in addition to a delay of 80-weeks before CJD diagnosis, with broad clinical manifestations, alterations in the MRI and the presence of protein 14-3-3 in CSF.

Although the patient had no reported family history of neurodegenerative or prion-related disorders, *PRNP* gene sequencing was not performed due to limited availability. Therefore, while the clinical and paraclinical findings strongly supported sporadic CJD, a hereditary prion disease cannot be definitively excluded.

In conclusion, not all the CJD symptoms are at the beginning of the disease, and not all the classical symptoms and signs indicate CJD. In addition, as seen in the review, the delay to a proper diagnosis of this disease is large, so this is a significant diagnostic challenge for physicians and neurologists. CJD should be included as a differential diagnosis at the beginning of behavioral symptoms or rapid cognitive impairment, even if it is a remote possibility.

## Authors’ contributions

Conceptualization: VEFR, YKYB, GAM, CAD, KPB; Data curation: VEFR, KPB; Investigation: VEFR, YKYB, GAM; Methodology: VEFR, ANF, CAD, KPB; Supervision: KPB; Writing – Original Draft Preparation: All authors; Writing – Review & Editing: All authors.

## Ethics and consent

This study was conducted according to the ethical standards of the Declaration of Helsinki. Informed consent was obtained from the patient and this case study was approved by the ethical board of Hospital de Lambayeque. Written informed consent for publication of their clinical details and/or clinical images was obtained from the patient.

## Data Availability

No data are associated with this article.
