# Supplementary material for: Case Report: Creutzfeldt-Jakob disease and diagnosis challenges: case report and evidence synthesis
Source: F1000Res. 2025 Dec 9;14:425. Originally published 2025 Apr 11. [Version 2] doi: 10.12688/f1000research.150498.2 (PMC12775658; doi:10.12688/f1000research.150498.2)
Supplement: Peer review history

# Reviewer report: Reviewer response for version 2

**DOI:** 10.5256/f1000research.192648.r439926

**Published:** 2026-01-06

**Authors:** Shi-Lin Yang

**Affiliations:** 1 Fudan University, Shanghai, China

I have no further comments to make.

Are enough details provided of any physical examination and diagnostic tests, treatment given and outcomes?

Yes

Is the case presented with sufficient detail to be useful for other practitioners?

Yes

Is sufficient discussion included of the importance of the findings and their relevance to future understanding of disease processes, diagnosis or treatment?

Yes

Is the background of the case’s history and progression described in sufficient detail?

Yes

Reviewer Expertise:

My major is cerebrovascular diseases and neuroophthalmology.

I confirm that I have read this submission and believe that I have an appropriate level of expertise to confirm that it is of an acceptable scientific standard.

# Reviewer report: Reviewer response for version 2

**DOI:** 10.5256/f1000research.192648.r439927

**Published:** 2025-12-30

**Authors:** Anna Ladogana

**Affiliations:** 1 Istituto Superiore di Sanità, Rome, Italy

 The manuscript has been comprehensively revised by the authors in accordance with our comments. All issues highlighted in the reviewer’s report have been duly addressed.

 The manuscript is hereby approved.

Are enough details provided of any physical examination and diagnostic tests, treatment given and outcomes?

No

Is the case presented with sufficient detail to be useful for other practitioners?

Partly

Is sufficient discussion included of the importance of the findings and their relevance to future understanding of disease processes, diagnosis or treatment?

Partly

Is the background of the case’s history and progression described in sufficient detail?

Partly

Reviewer Expertise:

prion diseases

I confirm that I have read this submission and believe that I have an appropriate level of expertise to confirm that it is of an acceptable scientific standard.

# Reviewer report: Reviewer response for version 1

**DOI:** 10.5256/f1000research.165074.r390131

**Published:** 2025-06-16

**Authors:** Shi-Lin Yang

**Affiliations:** 1 Fudan University, Shanghai, China

This article made a detailed review and summary of the previous literature, which is helpful to improve understanding of the CJD. The clinical manifestations of CJD are heterogeneous. Multiple case analyses have been reported. This article focused on reviewing the characteristics of the disease rather than more novel clinical manifestations.

Are enough details provided of any physical examination and diagnostic tests, treatment given and outcomes?

Yes

Is the case presented with sufficient detail to be useful for other practitioners?

Yes

Is sufficient discussion included of the importance of the findings and their relevance to future understanding of disease processes, diagnosis or treatment?

Yes

Is the background of the case’s history and progression described in sufficient detail?

Yes

Reviewer Expertise:

My major is cerebrovascular diseases and neuroophthalmology.

I confirm that I have read this submission and believe that I have an appropriate level of expertise to confirm that it is of an acceptable scientific standard, however I have significant reservations, as outlined above.

# Response

**Published:** 2025-11-29

**Authors:** Kevin Pacheco-Barrios

**Affiliations:** Universidad San Ignacio de Loyola, Lima District, Lima Region, Peru, Peru

**Reviewer 2: Shi-Lin Yang**

 This article made a detailed review and summary of the previous literature, which is helpful to improve understanding of the CJD. The clinical manifestations of CJD are heterogeneous. Multiple case analyses have been reported. This article focused on reviewing the characteristics of the disease rather than more novel clinical manifestations.

**Response.** Thank you for your appraisal. The manuscript has been updated based on the comments from the other reviewer. All changes can be observed in the new version with tracked changes.

 Is the background of the case’s history and progression described in sufficient detail?

 Yes

 Are enough details provided of any physical examination and diagnostic tests, treatment given and outcomes?

 Yes

 Is sufficient discussion included of the importance of the findings and their relevance to future understanding of disease processes, diagnosis or treatment?

 Yes

 Is the case presented with sufficient detail to be useful for other practitioners?

 Yes

# Reviewer report: Reviewer response for version 1

**DOI:** 10.5256/f1000research.165074.r384616

**Published:** 2025-06-14

**Authors:** Anna Ladogana

**Affiliations:** 1 Istituto Superiore di Sanità, Rome, Italy

The authors report a case of CJD with an atypical onset that initially made the diagnosis challenging. They also present a systematic review of the literature on published CJD case reports.

 My suggestions are the following

**Introduction**

Main issue

 The background does not include the most recent diagnostic criteria for CJD, In the 4th sentence "The probable diagnosis…" eliminate ref 1,3 and 5 and add see CDC diagnostic criteria and Hermann 2021 ECDC diagnostic criteria.

Minor

 I suggest

 First sentence:

 always fatal instead of " high mortality". 

 Second sentence:

 Sometime forming extracellular amyloid plaques instead of "forming extracellular amyloid plaques" and change the ref 2 with **Baiardi et al 2019**. 

 Third sentence:

 Add **Zerr et al 2024** in the references and delete references 3.

**Are enough details provided of any physical examination and diagnostic tests**

 Although the clinical picture and some test results are suggestive, extrapyramidal, pyramidal, cerebellar signs, or myoclonus are not clearly reported. In the absence of at least two of these symptoms, a diagnosis of probable CJD cannot be made.

 Regarding the diagnostic test for 14-3-3 protein using the Western blot method: how is a high titre measured?

 As for the brain MRI, the figure is not very clear—possibly due to motion artifacts. Please consider adding arrows to indicate the areas of high signal intensity.

 Given the relatively young age at onset, was family history investigated or genetic test for *PRNP gene *mutations done?

 The term of the search in Medline and Embase should be reported.

Minor

 This sentence should be checked, something seems missed: "The progressive neurological compromise with the development of early and progressive dementia in the next months…...

 CJD diagnostic was suspected"

**Is sufficient discussion included of the importance of the findings and their relevance to future understanding of disease processes, diagnosis or treatment**

 Again, there is a problem with the case classification if the authors did not report on other neurologic symptoms/signs as reported in the diagnostic criteria.

Are enough details provided of any physical examination and diagnostic tests, treatment given and outcomes?

No

Is the case presented with sufficient detail to be useful for other practitioners?

Partly

Is sufficient discussion included of the importance of the findings and their relevance to future understanding of disease processes, diagnosis or treatment?

Partly

Is the background of the case’s history and progression described in sufficient detail?

Partly

Reviewer Expertise:

prion diseases

I confirm that I have read this submission and believe that I have an appropriate level of expertise to confirm that it is of an acceptable scientific standard, however I have significant reservations, as outlined above.

## References

[1] : Creutzfeldt–Jakob disease and other prion diseases. *Nature Reviews Disease Primers* .2024;10(1) : 10.1038/s41572-024-00497-y 10.1038/s41572-024-00497-y 38424082

[2] : Recent advances in the histo‐molecular pathology of human prion disease. *Brain Pathology* .2019;29(2) : 10.1111/bpa.12695 278-300 10.1111/bpa.12695 PMC802868530588685

[3] : Biomarkers and diagnostic guidelines for sporadic Creutzfeldt-Jakob disease. *The Lancet Neurology* .2021;20(3) : 10.1016/S1474-4422(20)30477-4 235-246 10.1016/S1474-4422(20)30477-4 33609480 PMC8285036

# Response

**Published:** 2025-11-29

**Authors:** Kevin Pacheco-Barrios

**Affiliations:** Universidad San Ignacio de Loyola, Lima District, Lima Region, Peru, Peru

**Reviewer 1: Ana Ladogana**

- The authors report a case of CJD with an atypical onset that initially made the diagnosis challenging. They also present a systematic review of the literature on published CJD case reports.
  My suggestions are the following:

 

- **Introduction**
Main issue
  The background does not include the most recent diagnostic criteria for CJD, In the 4th sentence "The probable diagnosis…" eliminate ref 1,3 and 5 and add see CDC diagnostic criteria and Hermann 2021 ECDC diagnostic criteria.
  R. Thank you for your suggestions. The two references were added while the other three references were removed from the manuscript as suggested.

 

- **Minor**
  I suggest
  First sentence:
  always fatal instead of " high mortality". 

**Response.** Thank you for your suggestion. The sentences was rephrased.

  

- Second sentence:
  Sometime forming extracellular amyloid plaques instead of "forming extracellular amyloid plaques" and change the ref 2 with **Baiardi et al 2019**. 

**Response.** Thank you for your suggestion. We have rephrased the sentence accordingly.

  

- Third sentence:
  Add **Zerr et al 2024** in the references and delete references 3.
**Response.** Thank you for your suggestion. This reference was added to the text.

 

- **Are enough details provided of any physical examination and diagnostic tests**
  Although the clinical picture and some test results are suggestive, extrapyramidal, pyramidal, cerebellar signs, or myoclonus are not clearly reported. In the absence of at least two of these symptoms, a diagnosis of probable CJD cannot be made.

**Response.** We appreciate the reviewer’s important clarification regarding the diagnostic criteria for probable CJD. In our case, several neurological features consistent with pyramidal and extrapyramidal involvement were present, but we recognize that they were not described explicitly enough in the manuscript. As documented in the case narrative, the patient exhibited:

1. Extrapyramidal signs — including rest tremor, intention tremor, and parkinsonian-like motor slowing;
2. Pyramidal signs — such as hyperreflexia and bilateral Babinski sign noted during hospitalization (we added these details);
3. Cerebellar involvement — suggested by intention tremor and progressive gait impairment;
4. Episodes compatible with myoclonus — reported during clinical deterioration but not explicitly labeled as myoclonus in the original text.

To address the reviewer’s point, we have revised the clinical description to clearly and systematically report these neurological signs. This ensures alignment with the diagnostic criteria that require at least two of the following: myoclonus, visual/cerebellar signs, pyramidal/extrapyramidal signs, or akinetic mutism.

  

- Regarding the diagnostic test for 14-3-3 protein using the Western blot method: how is a high titre measured?

**Response.** We thank the reviewer for this question and agree that the description of the 14-3-3 Western blot method and the definition of a “high titre” (titer) should be clearer.

 In our case, the 14-3-3 protein was measured in CSF by a semi-quantitative Western blot assay performed in an external reference laboratory. The laboratory runs patient CSF samples alongside positive and negative controls. Band intensity corresponding to 14-3-3 is then densitometrically compared with these controls, and the result is reported using their internal semi-quantitative scale (e.g. negative, low, moderate, or high). In our patient, the sample was reported as “strongly positive / high titre”, meaning that the 14-3-3 band intensity was clearly above the laboratory’s predefined positivity threshold and comparable to or higher than their positive control. We have now clarified in the case description.

  

- As for the brain MRI, the figure is not very clear—possibly due to motion artifacts. Please consider adding arrows to indicate the areas of high signal intensity.
  R. Thank you, we have added the arrows in the figure as suggested.

 

- Given the relatively young age at onset, was family history investigated or genetic test for *PRNP gene *mutations done?

**Response.** We appreciate the reviewer’s important observation regarding early-onset cases and the possibility of hereditary prion disease. In our patient, no family history of neurodegenerative disorders, rapidly progressive dementia, psychiatric disease, or movement disorders was reported by relatives during the clinical assessment. Because the clinical presentation, evolution, MRI pattern, and 14-3-3 positivity were all compatible with sporadic CJD, and due to limited diagnostic resources at our institution, genetic testing for PRNP mutations was not performed. We agree that in younger patients genetic testing may be informative, and we have now acknowledged this point in the manuscript as a limitation. We specify that although no family history was identified, the absence of PRNP testing prevents us from fully excluding a genetic form of prion disease.

  

- Minor
  This sentence should be checked, something seems missed: "The progressive neurological compromise with the development of early and progressive dementia in the next months…...
  CJD diagnostic was suspected"
**Response.** Thank you for your comment. The sentence was corrected.

 

- **Is sufficient discussion included of the importance of the findings and their relevance to future understanding of disease processes, diagnosis or treatment**
  Again, there is a problem with the case classification if the authors did not report on other neurologic symptoms/signs as reported in the diagnostic criteria.

- **Is the background of the case’s history and progression described in sufficient detail?**

Partly

- **Are enough details provided of any physical examination and diagnostic tests, treatment given and outcomes?**

No

- **Is sufficient discussion included of the importance of the findings and their relevance to future understanding of disease processes, diagnosis or treatment?**

Partly

- **Is the case presented with sufficient detail to be useful for other practitioners?**

Partly

**References**

 1. Zerr I, Ladogana A, Mead S, Hermann P, et al.: Creutzfeldt–Jakob disease and other prion diseases. *Nature Reviews Disease Primers*. 2024; **10** (1). Publisher Full Text

 2. Baiardi S, Rossi M, Capellari S, Parchi P: Recent advances in the histo‐molecular pathology of human prion disease. *Brain Pathology*. 2019; **29** (2): 278-300 Publisher Full Text

 3. Hermann P, Appleby B, Brandel J, Caughey B, et al.: Biomarkers and diagnostic guidelines for sporadic Creutzfeldt-Jakob disease. *The Lancet Neurology*. 2021; **20** (3): 235-246 Publisher Full Text
